# RSDNet: A New Multiscale Rail Surface Defect Detection Model

**DOI:** 10.3390/s24113579

**Published:** 2024-06-01

**Authors:** Jingyi Du, Ruibo Zhang, Rui Gao, Lei Nan, Yifan Bao

**Affiliations:** College of Electrical and Control Engineering, Xi’an University of Science and Technology, Xi’an 710054, China; 000248@xust.edu.cn (J.D.); gaorui@xust.edu.cn (R.G.); 22206223076@stu.xust.edu.cn (L.N.); 23206223101@stu.xust.edu.cn (Y.B.)

**Keywords:** rail surface defect detection, YOLOv8, CDConv, BiFPN, EMA

## Abstract

The rapid and accurate identification of rail surface defects is critical to the maintenance and operational safety of the rail. For the problems of large-scale differences in rail surface defects and many small-scale defects, this paper proposes a rail surface defect detection algorithm, RSDNet (Rail Surface Defect Detection Net), with YOLOv8n as the baseline model. Firstly, the CDConv (Cascade Dilated Convolution) module is designed to realize multi-scale convolution by cascading the cavity convolution with different cavity rates. The CDConv is embedded into the backbone network to gather earlier defect local characteristics and contextual data. Secondly, the feature fusion method of Head is optimized based on BiFPN (Bi-directional Feature Pyramids Network) to fuse more layers of feature information and improve the utilization of original information. Finally, the EMA (Efficient Multi-Scale Attention) attention module is introduced to enhance the network’s attention to defect information. The experiments are conducted on the RSDDs dataset, and the experimental results show that the RSDNet algorithm achieves a mAP of 95.4% for rail surface defect detection, which is 4.6% higher than the original YOLOv8n. This study provides an effective technical means for rail surface defect detection that has certain engineering applications.

## 1. Introduction

During the train operation, frequent collisions between wheels and rails and the factor of outdoor environmental erosion can easily lead to defects on the rail surface, which may cause serious accidents if not handled in time [1]. The initial stage of track defect detection mainly adopts the inspection method; however, this method is inefficient and easily affected by subjective factors [2]. With the development of sensor and communication technologies, fault detection based on the dynamic response of wheels and rails has been widely used. Rail inspection has shifted to the use of sensors and automated equipment such as ultrasonic detection and eddy current detection [3,4,5,6,7]. This method uses sensors to capture vibration and acceleration signals during operation and analyzes these signals to identify abnormalities in the wheel-rail system and determine faults [8]. Fu et al. [9] simulate flatness anomalies using the multi-body dynamics software SIMPACK and generate spectral images for anomaly detection by analyzing acceleration signals. Xie et al. [10] developed a vehicle-track coupled dynamics model to simulate the dynamic response of the axle box under different speeds and track wear excitations. The sensor-based method has low environmental requirements and a small cost [11,12,13]. However, it may not be sensitive enough to detect subtle faults in some cases (e.g., surface cracks or minor wear), and it also affects the accuracy of detection in complex environments, such as when there is a large amount of noise interference. At the same time, the sensors mainly detect vibration and do not provide a visual image of the fault, which is not intuitive enough in some cases where quick diagnosis and repair are required.

In recent years, machine vision detection has been widely used in rail surface defect detection due to its accurate, rapid, and non-contact characteristics [14]. Machine vision detection is categorized into traditional image processing methods and deep learning detection methods as per the development time [15]. Traditional image processing methods require manually designed features or predefined defect features. The defects in the image are identified and localized by classifier settings, morphological operations, etc. [16]. Gan et al. [17] localized the railroad surface image defects through a two-stage algorithm, where the rough extractor initially locates the defects in the railroad surface image and the detail extractor further determines whether the anomalies are real defects or not. Zhang et al. [18] used a curvature filter to extract the structural information of the rail surface and used an improved Gaussian mixture model to identify the defects. Nieniewski [19] employed morphological operations such as corrosion and expansion to highlight defective features on the rail surface and identified defective areas in the image through setting thresholds and conditions. However, traditional machine learning detection methods are sensitive to noise in images and have poor generalization ability, which limits their application in real-rail defect detection.

With the rapid development of deep learning, convolutional neural networks have become an obvious choice for defect detection on rail surfaces due to their unique feature representation advantages and modeling capabilities. Based on the utilization of region proposal networks, deep learning-based object detection methods are classified into two-stage or one-stage networks [20]. Typical algorithms for two-stage target measurement include R-CNN [21], Faster-RCNN [22], and Mask-RCNN [23], etc. These algorithms utilize RPN to quickly generate and screen out candidate regions containing rail surface defects at the initial stage, providing a basis for subsequent defect classification and localization. Yu et al. [24] proposed the method of migration learning to train the network to realize rail surface defect detection. Bai et al. [25] used faster R-CNN to classify and detect the labeled image dataset and enhanced the detection rate and accuracy by optimizing the anchor box function. Wang et al. [26] designed a multi-scale feature pyramid based on a two-stage network to adapt to the detection of track defects of different sizes, and the CIOU evaluation metrics were also introduced to optimize the performance of the RPN to achieve the precise location of the defects. Although the two-stage inspection method has advantages in accuracy, it still faces challenges such as inaccurate candidate region generation, slow detection speed, and difficulty in recognizing small-scale defects in practical applications.

One-stage target detection methods use an end-to-end approach to accomplish the target detection task directly without generating candidate boxes, and the typical algorithms for single-stage target measurement are the YOLO (You Only Look Once) series [27,28,29,30,31,32], SSD (Single Shot MultiBox Detector) [33], etc., which have the advantages of simplicity and high efficiency, etc. [34]. The YOLO series offers significant advantages in terms of fast response, efficient deployment, and adaptability, and more and more researchers are using YOLO algorithms in rail surface defect detection. Wang et al. [35] designed spatial attention sharpening filters based on YOLOv5s to enhance attention to the defects at the edge location of the rail defects and constructed M-ASFF to enhance the details of the underlying features of tiny defects. Zhang et al. [36] used BiFPN for feature fusion at the neck of YOLOX and also fused the NAM attention mechanism to improve the image feature expression ability, and the experimental results showed that the defect recognition rate was improved by 2.42% compared to YOLOX. Wang et al. [37] addressed the problem of detecting small targets and dense occlusion on the surface of rails by introducing the SPD-Conv building block in YOLOv8 to improve detection attention to small and medium-sized targets, and the Focal-SIoU loss function was used to adjust the sample weights to improve the model’s ability to recognize complex samples. The YOLOv8 network is one of the newest open-source neural networks in the YOLO family, offering high performance in terms of detection speed and accuracy [38].

In conclusion, to address the issue of rail surface defects with different scales and many small-scale defects, this study proposes a track surface defect detection algorithm, RSDNet, based on the improved YOLOv8 algorithm. The primary contributions of this study are summarized as follows:

(1) Proposed CDConv (Cascade Dilated Convolution), a module based on feature reuse. It was introduced into Backbone to realize multi-scale feature extraction without increasing the number of too many parameters.
(2) Based on the idea of BiFPN (Bi-directional Feature Pyramids Network), change the feature fusion method of Head, add jump connections, and utilize more original feature information for feature fusion to improve the network’s ability to recognize defective edges.
(3) Incorporate the EMA (Efficient Multi-Scale Attention) module into Head to enhance the feature extraction network’s attention to defect detail information, thus improving the detection accuracy of rail surface defects.

## 2. YOLOv8

**Figure 1:**
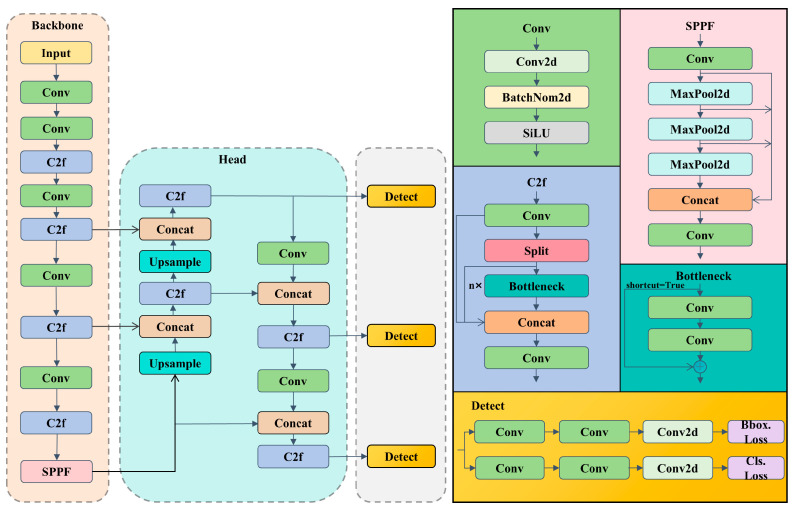
The structure of YOLOv8.

YOLOv8 is a one-stage target detection algorithm proposed by Ultralytics in 2023. Its performance is so superior that it outperforms most of the target detection algorithms. Therefore, YOLOv8 is the baseline model chosen in this study. YOLOv8 is mainly composed of the Backbone, Head, and Detector, and its structure is shown in Figure 1.

YOLOv8 adjusts the input image to 640 × 640 resolution. The Backbone consists of CBS, C2f, and SPPF modules. The CBS module includes Conv, BatchNormal, and SiLU, which realize the transformation and extraction of the input features; the C2f module captures the gradient flow information by using Bottleneck units; and the SPPF module reduces the computation and improves the feature extraction efficiency through the serial stacked pooling layer to reduce the computation and enhance the feature extraction efficiency. Head adopts the PANet structure to realize feature fusion and information transfer. Detect uses a decoupled Head to separate the regression and prediction branches. Through the DFL (Distribution Fusion Loss) strategy, the regression coordinates are regarded as distributions rather than single values, which helps the model deal with the defects of small scales or irregular shapes in a way that provides more accurate localization information.

YOLOv8n, as the smallest model in the YOLOv8 series, has the advantages of fast detection speed and low resource consumption. However, if YOLOv8n is directly applied to the task of detecting defects on the rail surface, the model will face challenges such as diverse defect scales and more small-scale defects. In order to solve these problems, targeted adjustments to the model are needed to enhance the model’s ability to recognize targets at different scales, thereby improving the overall performance of track surface defect detection.

## 3. RSDNet: YOLOv8n-CDConv-BiFPN-EMA

**Figure 2:**
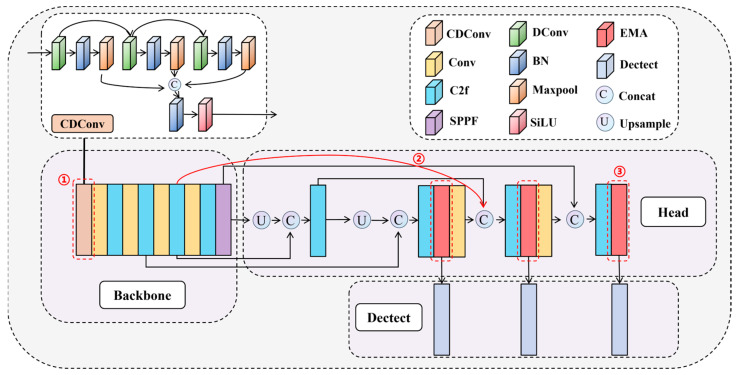
The structure of the proposed method, RSDNet. (YOLOv8n-CDConv-BiFPN-EMA).

RSDNet is based on the YOLOv8n model, and the designed CDConv module is introduced in Backbone to realize multi-scale feature extraction. Drawing on the BiFPN idea in Head, a new fusion method is designed to enhance the network’s utilization of raw information. Meanwhile, EMA is fused to enhance attention to the defect information. Figure 2 illustrates the architectural design of the RSDNet model. Where ① denotes the designed CDConv module, ② denotes the designed feature fusion method, and ③ denotes the location where EMA is added.

Compared with YOLOv8, the improved algorithm is able to capture long-distance dependencies in the image earlier, fuse more low-level semantic information such as defect edges, and dynamically emphasize and guide key defect detail information to achieve more accurate rail surface defect detection.

### 3.1. CDConv Module Proposed in This Study

**Figure 3:**
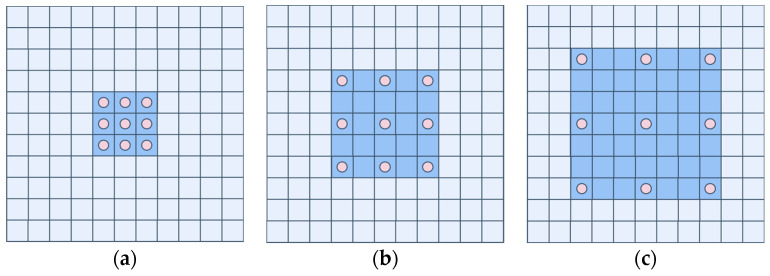
Comparison between Regular Convolution and Dilated Convolution. (**a**) is a regular convolution process (dilation rate = 1), and the receptive field is 3; (**b**) is the dilated convolution with dilation rate = 2, and the receptive field is 5; (**c**) is the dilated convolution with dilation rate = 3, and the receptive field is 7.

Dilated convolution is a convolution technique proposed by Google in 2015 for enlarging the receptive field, which was first applied to the DeepLab model [39]. Dilated convolution is able to increase the receptive field without increasing the number of parameters as shown in Figure 3, the same 3 × 3 convolution kernel can have the effect of 5 × 5 and 7 × 7 convolution.

**Figure 4:**
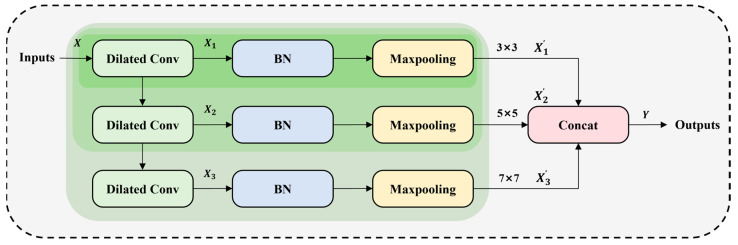
The structure of the Cascaded Dilated Convolution (CDConv).

When detecting defects on the rail surface, the direct use of multiple cavity convolutions tends to increase too many parameters, although it can increase the receptive field. The CDConv module designed in this paper adopts a feature reuse strategy to realize parameter sharing by connecting multiple cavity convolution layers in series, which reduces the consumption of computational resources. In addition, the CDConv module realizes multi-scale feature extraction of the input defective image by setting multiple parallel branches with different receptive fields. The CDConv module is shown in Figure 4.

Assume the input feature mapping is X. With three cascading null convolutions, it can be obtained as:

(1) $$X_{1}=D_{1}\left(X\right),$$

(2) $$X_{2}=D_{2}\left(X_{1}\right),$$

(3) $$X_{3}=D_{3}\left(X_{2}\right),$$

where $D_{i}\left(\cdot \right)$ is a dilated convolution operation with a specific dilated rate.

Next, the output of each dilated convolution is subjected to BatchNormal $\left(BN\right)$ and Maximum Pooling $\left(Maxpooling\right)$ layers to further optimize the feature representation and speed up the computational process. These operations can be represented as:

(4) $$X_{1}^{\prime }=Max\left(BN\left(X_{1}\right)\right),$$

(5) $$X_{2}^{\prime }=Max\left(BN\left(X_{2}\right)\right),$$

(6) $$X_{3}^{\prime }=Max\left(BN\left(X_{3}\right)\right),$$

where $BN\left(\cdot \right)$ and $Max\left(\cdot \right)$ denote the operations of BN and Maxpooling, respectively, and $X_{i}^{\prime }$ are the outputs after BatchNormal and Maximum Pooling.

By connecting these three output feature maps, a feature representation containing multi-scale information can be obtained.

(7) $$Y=\left[X_{1}^{\prime },X_{2}^{\prime },X_{3}^{\prime }\right],$$

Aiming at the problem that small target defects occupy few pixels in the image, this study applies CDConv to the first layer after the input of the YOLOv8n model so that the model can maintain a higher resolution from the beginning, and improve the model’s ability to recognize and localize the small target defects in the initial stage. Meanwhile, the flexible feature extraction capability of CDConv can better adapt to various shape defects that may appear in complex scenes, making the model more robust.

### 3.2. Feature Extraction Method in This Study

**Figure 5:**
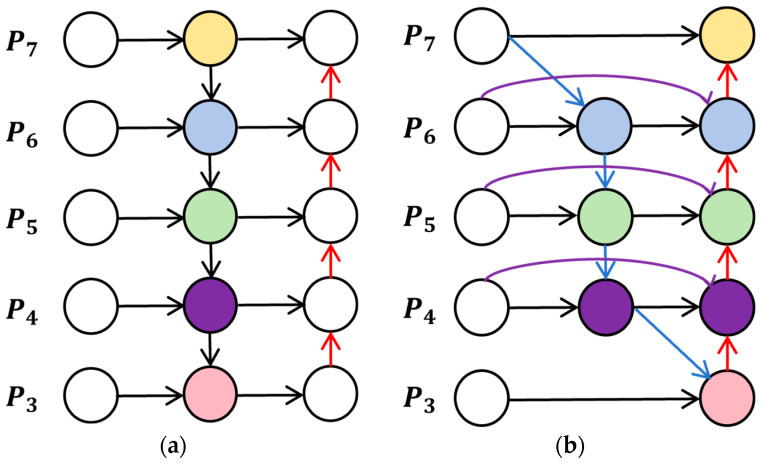
Feature network design. (**a**) PANet; (**b**) BiFPN.

BiFPN is a weighted bidirectional feature pyramid network proposed by Mingxing Tan et al. in EffiicientDet [40]. BiFPN facilitates the flexibility and effectiveness of the information flow of feature maps between different layers by introducing weighted bidirectional connections. Compared to the fusion approach originally adopted by YOLOv8n, BiFPN realizes cross-scale connectivity with the design changes shown in Figure 5.

In the design of the YOLOv8n network, the feature fusion strategy employs an optimized PANet structure to enhance feature integration efficiency. However, this fusion method fails to fully exploit the potential of the original feature information. To further improve the performance of the model, this study draws on the idea of BiFPN to improve the feature fusion mechanism.

**Figure 6:**
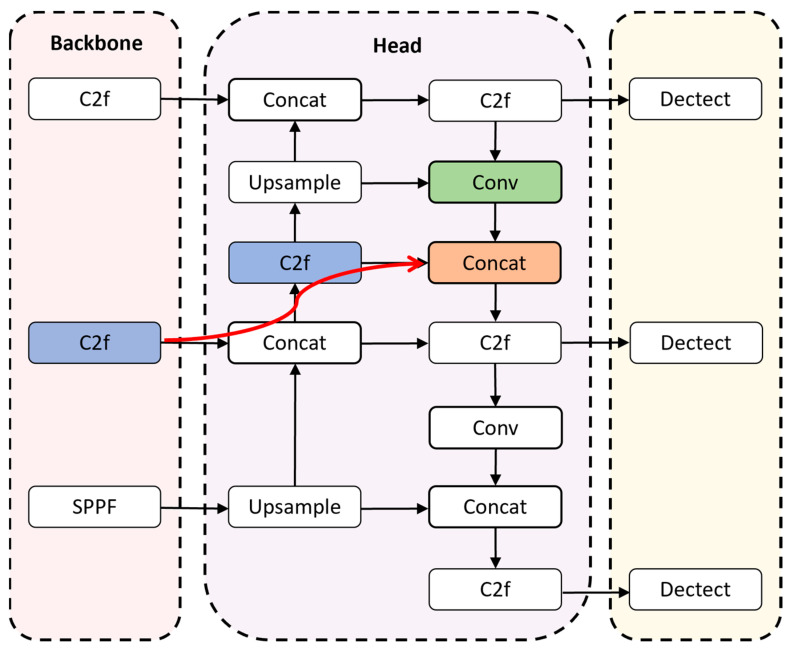
The structure of feature fusion.

The improved feature fusion is shown in Figure 6. After the third C2f layer of the backbone, a new path is introduced to fuse the features extracted from this layer with the features from the first C2f layer of the Head part and the first Conv layer of the neck part. This design makes the raw, detail-rich features that have not been multiprocessed participate more in the feature fusion process, reduces the loss of information in the transfer process, and makes the model capture the defect detail information more acutely.

Since different input features have different resolutions, they usually contribute unequally to the output features. To solve this problem, BiFPN adds an extra weight to each input and allows the network to learn the importance of each input feature. Normalized fusion is shown in Equation (8), which is less computationally intensive and has similar accuracy compared to Softmax function-based fusion methods.

(8) $$OUT=\sum_{i}\frac{w_{i}}{\varepsilon +\sum_{j}w_{j}}\cdot IN_{i},$$

where $w_{i}$ is a learnable weight that can be a scalar (per feature), a vector (per channel), or a multidimensional tensor (per pixel).

By fusing more feature information and improving the utilization of the original feature information, the generalization ability of the model and the recognition ability of the defect edge information are improved. The weighting mechanism also ensures that the importance of different levels of features is reasonably balanced, which helps to improve the model’s ability to detect defects in small targets.

### 3.3. Head Network with EMA

**Figure 7:**
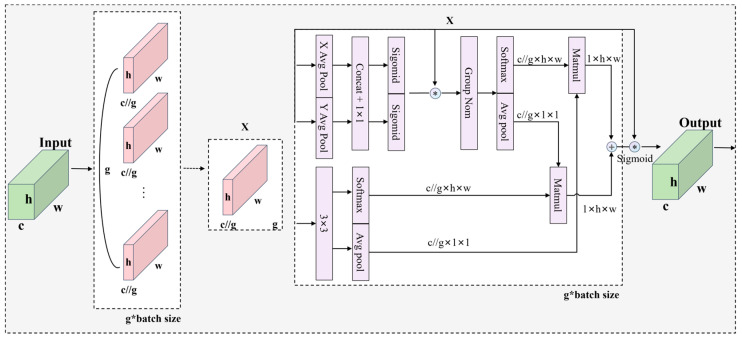
EMA mechanism structure diagram.

Due to the complex and variable background of the track surface and the difficulty in detecting subtle defects, the EMA attention mechanism is introduced in order to further enhance the screening and filtering abilities of the network on key information and improve the performance of the algorithm. EMA is a cross-space learning approach proposed by Daliang Ouyang et al., Efficient Multi-Scale Attention, that can interact with information without channel dimensionality reduction and reduce computational overhead [41]. Its structure is shown in Figure 7. In this image + indicates an addition operation and * indicates a multiplication operation.

The EMA module divides the input feature map X along the channel dimension into G groups, each of which can be represented as $X_{i}$. Each sub-feature group is learned to obtain the corresponding weights, allowing the network to focus on different regions and features in the track surface image.

(9) $$X_{i}={\left\{X_{i,j}\right\}}_{j=1}^{C/G},$$

where i is the index of the group, C is the total number of input channels, and G is the number of subgroups.

For each group $X_{i}$, EMA employs two parallel branches to capture cross-dimensional interactions capturing pixel-level relationships, and improved feature representation, the outputs of which can be denoted as $F_{1\times 1}$ and $F_{3\times 3}$. For the outputs of the 1 × 1 branch and the 3 × 3 branch, the channel weights are adjusted using 2D global average pooling coding, respectively.

(10) $$P_{1\times 1}=\frac{1}{H\times W}\sum_{i=1}^{H}\sum_{j=1}^{W}F_{1\times 1}\left(i,j\right),$$

(11) $$P_{3\times 3}=\frac{1}{H\times W}\sum_{i=1}^{H}\sum_{j=1}^{W}F_{3\times 3}\left(i,j\right),$$

Finally, the information from these two branches is fused by a matrix dot product operation to get the final output feature map $X_{EMA}$.

(12) $$X_{EMA}=\sigma \left(P_{1\times 1}\cdot P_{3\times 3}+X\right),$$

where σ is the Sigmoid activation function,⋅ denotes the matrix dot product operation, and X is the original input feature map. The matrix operation captures pixel-level relationships while avoiding the lack of channel information richness due to dimensionality reduction.

**Figure 8:**
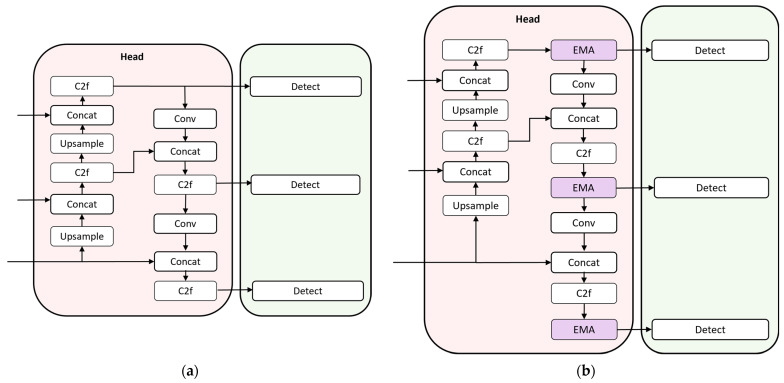
Schematic of the location where the EMA module is added. (**a**) The structure of the Head; (**b**) The structure of added EMA Head.

As shown in Figure 8, the EMA is combined with the three C2f connecting Detect in the Head. The EMA combines the C2f to ensure the full utilization of the features at each scale. The cross-space learning mechanism of the EMA is able to aggregate defect feature information from different branches. At the same time, EMA dynamically adjusts the weights in such a way that it can strengthen the key defect regions in the track surface feature map and ignore irrelevant background information, so as to improve the accuracy of track surface defect detection.

## 4. Experiments

### 4.1. Experimental Details

#### 4.1.1. Dataset

**Figure 9:**
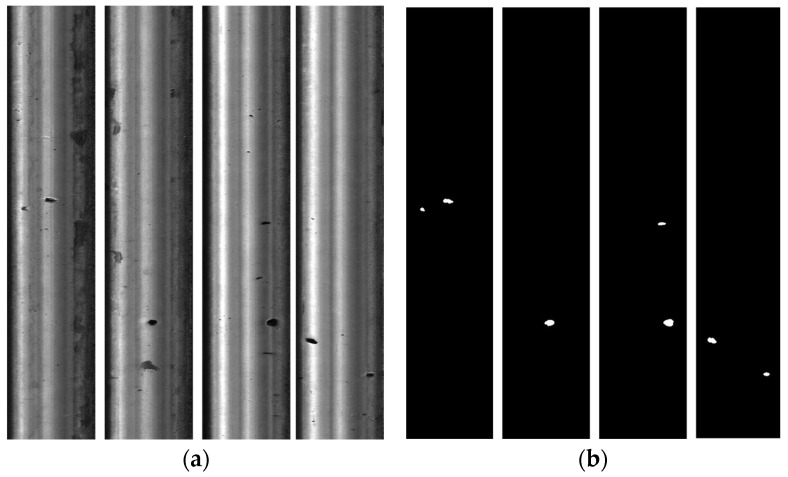
Examples of RSDD datasets. (**a**) Rail surface images; (**b**) GroundTruth.

In this study, the RSDDs dataset collected and made publically available by Beijing Jiaotong University was used, as shown in Figure 9. The dataset contains 195 difficult rail surface defect photos, with 67 being 160 pixels × 1000 pixels and 128 being 55 pixels × 1250 pixels. After segmentation and image adjustment screening, 240 track surface defect images were obtained, and an initial track surface defect image sample dataset was established. Then, based on the initially established dataset, the dataset is expanded using the flip transform, random cropping, and brightness transform. Finally, 1080 rail surface defect images are generated, in which the ratio of the training set, validation set, and test set is 7:2:1.

#### 4.1.2. Experimental Environment

Experimental and Parameter Configuration The experimental setup is shown in Table 1. For model training, this study used an Intel(R) Xeon(R) CPU E5-2640 v4 (Intel Corporation, Santa Clara, CA, USA) and an NVIDIA Quadro P2200 GPU (NVIDIA Corporation, Santa Clara, CA, USA). The software environment consisted of CUDA version 10.2, Python 3.8, and Pytorch version 2.2.1.

#### 4.1.3. Evaluation Index

In this study, precision (P), recall (R), and mean average precision (mAP) were used as metrics to assess the effectiveness of the algorithm for detection. Among them, mAP comprehensively evaluates the performance of the model in detecting all categories. The following are the specific calculations of the evaluation metrics:

(13) $$P=\frac{TP}{TP+FP},$$

(14) $$R=\frac{TP}{TP+FN},$$

where P denotes the prediction precision of the model and R denotes the recall of the model. TP represents the number of correctly classified positive samples, FP represents the number of misclassified positive samples and FN represents the number of misclassified negative samples.

(15) $$AP=\int_{0}^{1}P\left(R\right)dR,$$

(16) $$mAP=\frac{1}{n}\sum_{i=1}^{n}AP_{i},$$

where AP represents the area under the precision-recall curve for a particular category at different confidence thresholds. mAP stands for mean accuracy and is the average of the APs for each category.

### 4.2. Results and Discussion

#### 4.2.1. Ablation Experiments

In this paper, ablation experiments of CDConv, BiFPN, and EMA attention mechanisms were performed sequentially. The results of the experiments are shown in Table 2.

The first row of data in Table 2 shows the performance of the original YOLOv8n model without any improvements, where the average accuracy on the rail surface defect detection task is 90.8%. To further improve the performance of the model, the convolution operation for the Backbone and the fusion method for the Head have been improved. In the second and third rows of the table, the model performance after introducing the CDConv module and adopting the BiFPN fusion method is shown, respectively. By adding the CDConv module, the model’s mAP on rail face defect detection is improved to 94.1%, and the multi-scale feature extraction capability of CDConvd enhances the model’s detection capability. The mAP also reaches 92.3% with BiFPN fusion, which is due to BiFPN’s ability to integrate global and contextual information more effectively. Finally, the EMA attention mechanism is introduced in the Head section, which significantly improves the accuracy of the model, even though this improvement leads to only a slight increase in the number of parameters. The EMA module further enhances the detection performance of the model by focusing on hinge defect features.

**Figure 10:**
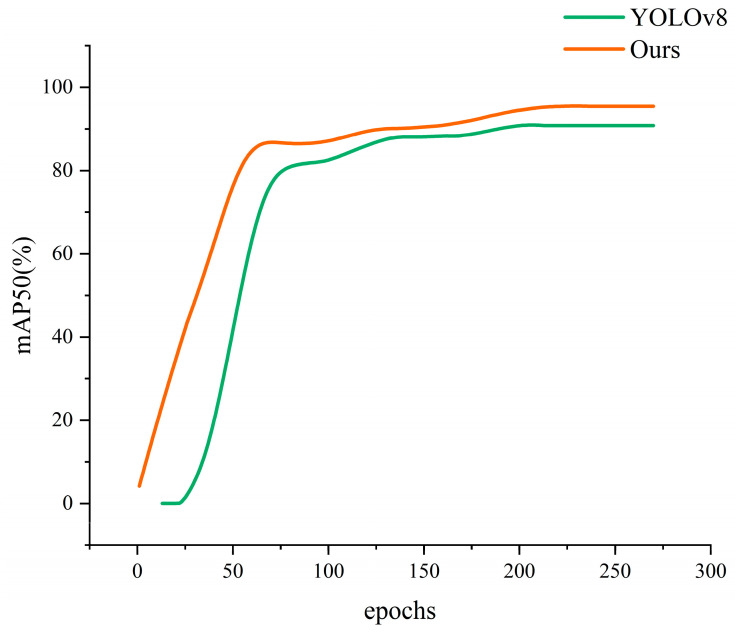
The mAP curves for the original YOLOv8 and the RSDNet.

The mAP is the area enclosed by the mapping of precision and recall on the two axes. In the experiments in this paper, the mAP curves of the initial YOLOv8n model and the improved YOLOv8n model are shown in Figure 10. These curves depict the performance trend of the two models during the training process. As can be seen from the figure, the mAP curves of the improved YOLOv8n show a clear upward trend throughout the training process, which indicates that the performance of the model is steadily improving with the training. After sufficient training, the mAP curve finally stabilizes at a higher plateau, indicating that the model’s performance has reached its peak. The mAP of the original YOLOv8 is 90.8%, while the mAP of this paper is 95.4%, which is 4.6% higher.

#### 4.2.2. Improved Model Comparison Experiments

This section experiments with CDConv, EMA position, and quantity in turn.

- Location and number of CDConv

Table 3 represents the effect of changing different numbers of Conv to CDConv at a variety of locations in the network model on the experimental results.

As can be seen from Table 3, adding CDConv modules to the backbone part of the network can effectively improve the detection performance of the model, especially when adding 1 module. With the increase in the number of CDConv modules, although the recall of the model has improved to some extent, accuracy and mAP@0.5 growth trends have leveled off or even decreased, which may be due to the overfitting caused by the increase in the model complexity. Adding CDConv modules to the Head part of the network also helps to improve the model performance, but the improvement is more limited compared to the backbone part. With this in mind, the first convolutional layer of Backbone is replaced with CDConv in this paper.

- Location and number of the EMA

Table 4 represents the effect of adding different numbers of EMAs at a variety of locations in the network model on the experimental results.

From Table 4, it can be seen that although EMA can effectively improve the accuracy of the backbone network part, the improvement is not as obvious as that of the Head. This may be related to the role of EMA in different network layers; Head, as the feature fusion layer in the model, serves to integrate the feature maps of different layers to provide rich contextual information for Dectect, while applying EMA in Head is more helpful for smoothing and stabilizing these features. Considering this, this paper adds EMA modules after the three C2f’s connected to Dectect in the Head.

#### 4.2.3. Performance Comparison Experiments

The performance of the improved algorithm is compared with several other typical target detection models in this work.

As shown in Table 5, the algorithm significantly outperforms the traditional Faster R-CNN and SSD algorithms for rail surface defect detection. Compared with the YOLOv5s, YOLOv7-Tiny, and YOLOv8n models in the YOLO family, the improved algorithm achieves 5.6%, 7.4%, and 4.6% on mAP@0.5. The inference time is 2.5 ms faster than YOLOv5s, but 0.4 ms and 1.3 ms slower than YOLOv7-Tiny and YOLOv8n, respectively. In addition, the algorithm also demonstrates high levels of accuracy and recall, two key performance metrics. These results fully validate the accuracy and stability of the RSDNet model in the task of rail surface defect recognition, which meets the practical needs of rail inspection.

**Figure 11:**
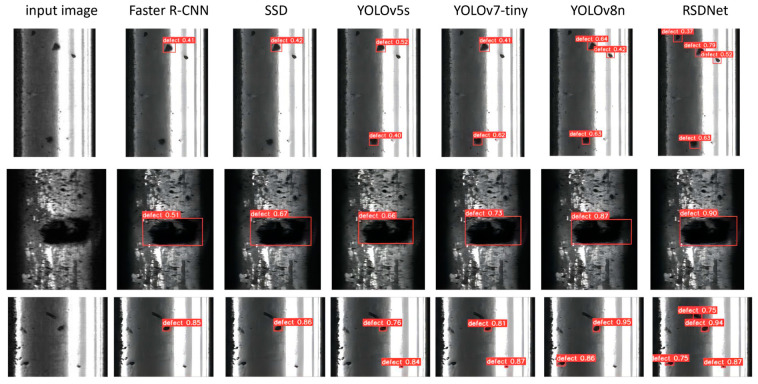
Comparison of the Detection Effect of Each Algorithm.

Figure 11 compares the performance of YOLOv5, YOLOv7-Tiny, YOLOv8n, Faster R-CNN, SSD, and optimized YOLOv8n for defect detection in this paper. As can be seen from the figure, Faster R-CNN and SDD have missed detection when the defective image is small, as shown in the first and third rows of the example in Figure 11, where the two algorithms fail to detect the tiny defects located in the lower-left corner of the image. Although YOLOv5s and YOLOv7-tiny could recognize most of the defects, they suffer from low detection accuracy, as shown in the second row of Figure 11, with a detection value of less than 0.75. Also miss-detection occurs when the defects are located in a complex background environment. This may be because these models are designed with limited detection capability for small targets or because they are not robust enough when dealing with complex backgrounds. In contrast, the optimized YOLOv8n algorithm proposed in this paper performs well in the defect detection task, not only capturing all defects in the image comprehensively but also significantly improving the accuracy of the bounding box prediction. This indicates that the optimized YOLOv8n improves the ability to identify and locate defects through algorithmic improvements while maintaining the fast processing of the YOLO series of algorithms.

## 5. Conclusions

In this study, an advanced multi-scale rail surface defect detection model, RSDNet, is proposed to address the challenges of significant scale differences and numerous small-scale defects in the detection of rail surface defects. The essence of RSDNet’s design lies in its excellent ability to capture multi-scale features, a feature that is crucial for accurately identifying small defects on the rail surface.

Based on YOLOv8n, RSDNet enhances the recognition of defects by designing the CDConv module, borrowing the BiFPN feature fusion approach, and introducing the EMA attention mechanism. Through these innovative improvements, RSDNet shows higher precision and recall in the task of track surface defect detection, and the algorithm proposed in this paper has higher accuracy, recall, and confidence in track surface defect recognition compared with other similar models. The multi-scale feature model performs well in terms of localization precision and detection precision, which can meet the practical requirements of real-time detection of track surface defects.

Looking ahead, the research team plans to collect more images of railroad track surface defects to enrich our dataset and refine the detailed descriptions of the defects. In addition, algorithms will be refined for images taken in strong-noise environments and blurred images that may arise from high-speed shooting to improve the generalization ability and robustness of the model. Efforts are made to apply the algorithms to embedded devices to detect defects on track surfaces in real time.

## Figures and Tables

**Table 1:** Experimental environment configuration.

Experimental Component	Version
CPU	Intel(R) Xeon(R) CPU E5-2640 v4
GPU	NVIDIA Quadro P2200
CUDA version	10.2
Python version	3.8
Pytorch version	2.2.1

**Table 2:** Comparison of results of ablation experiments.

Model	Parameter (M)	P (%)	R (%)	mAP@0.5 (%)
YOLOv8n	**30.06**	92.3	86.7	90.8
YOLOv8n+CDConv	30.11	96.0	88.6	94.1
YOLOv8n+BiFPN	30.22	95.6	87.3	92.3
YOLOv8n+EMA	30.17	95.8	87.9	93.7
YOLOv8n+CDConv+BiFPN	30.28	96.2	88.5	94.8
YOLOv8n+BiFPN+EMA	30.36	95.9	88.3	94.4
YOLOv8n+CDConv+BiFPN+EMA	30.41	**96.4**	**90.6**	**95.4**

**Table 3:** Effect of the position and number of CDConv on the model.

Model	Position	Numbers	P (%)	R (%)	mAP@0.5 (%)
YOLOv8n+CDConv	Backbone	1	95.4	88.9	94.1
YOLOv8n+CDConv	Backbone	3	94.7	88.4	93.9
YOLOv8n+CDConv	Backbone	5	95.2	88.6	91.4
YOLOv8n+CDConv	Head	1	94.3	88.6	88.8
YOLOv8n+CDConv	Head	2	94.4	88.5	89.4

**Table 4:** Effect of the position and number of EMA on the model.

Model	Position	Numbers	P (%)	R (%)	mAP@0.5 (%)
YOLOv8n+EMA	Backbone	1	94.2	86.5	90.9
YOLOv8n+EMA	Backbone	3	95.4	87.1	91.9
YOLOv8n+EMA	Backbone	4	95.2	87.6	92.5
YOLOv8n+EMA	Head	3	95.8	87.9	93.7
YOLOv8n+EMA	Head	4	94.6	87.5	93.2

**Table 5:** The results of comparison experiments.

Model	P (%)	R (%)	mAP@0.5 (%)	Times/ms
Faster R-CNN	71.5	72.9	72.3	156.5
SSD	78.3	67.0	75.1	46.2
YOLOv5s	86.7	80.8	89.8	22.3
YOLOv7-tiny	85.7	83.3	88.0	19.4
YOLOv8n	92.3	87.7	90.8	18.5
Ours	**96.4**	**90.6**	**95.4**	19.8

## Data Availability

The rail surface dataset in this paper is a homemade dataset and is not disclosed due to its use in subsequent studies.
